# Efficacy of an app-based multimodal lifestyle intervention on body weight in persons with obesity: results from a randomized controlled trial

**DOI:** 10.1038/s41366-023-01415-0

**Published:** 2023-11-28

**Authors:** Kathrin Gemesi, Stefanie Winkler, Susanne Schmidt-Tesch, Florian Schederecker, Hans Hauner, Christina Holzapfel

**Affiliations:** 1 Institute for Nutritional Medicine, School of Medicine & Health, Technical University of Munich, Munich, Germany; 2 Chair of Epidemiology, Department of Sport and Health Sciences, School of Medicine & Health, Technical University of Munich, Munich, Germany; 3 Else Kröner Fresenius Center for Nutritional Medicine, ZIEL – Institute for Food and Health, Technical University of Munich, Freising, Germany; 4 Department of Nutritional, Food and Consumer Sciences, Fulda University of Applied Sciences, Fulda, Germany

**Keywords:** Weight management, Public health

## Abstract

**Background:**

Despite an increasing number of smartphone applications (apps) addressing weight management, data on the effect of app-based multimodal obesity treatment approaches on weight loss is limited. This study aimed to examine the effect of a digital multimodal weight loss intervention program delivered by an app on body weight in persons with obesity.

**Methods:**

For this single-centre randomized controlled study, 168 adults with a body mass index (BMI) between 30.0 and 40.0 kg/m^2^ without severe comorbidities were recruited in the region of Munich and randomized into two intervention groups. The ADHOC group received an app-based multimodal weight loss program from baseline on for 12 weeks plus 12 weeks of follow-up. The EXPECT group received the app-based intervention for 12 weeks after 12 weeks of “waiting” (no intervention). Anthropometric data, data on quality of life (*EuroQol*, EQ-5D-5L), and app usage data were collected.

**Results:**

64.3% of study participants were women, mean age was 46.8 ± 11.0 years, and mean BMI was 34.2 ± 2.8 kg/m^2^. The completers analysis resulted in a weight loss of 3.2 ± 3.2 kg (3.2 ± 3.0%) in the ADHOC group and 0.4 ± 2.6 kg (0.3 ± 2.6%) in the EXPECT group after 12 weeks, with a significant difference between the groups (β [95% CI] = −2.9 [−3.8; −1.9], *p* < 0.001). Completers in the ADHOC group showed weight maintenance after 24 weeks. The time spent on the app was associated with weight reduction (β [95% CI] = −0.10 [−0.18; −0.01], *p* = 0.03).

**Conclusions:**

Application of a multimodal app-based weight loss program results in moderate weight loss in persons with obesity.

**Trial registration:**

This study was registered in the German Clinical Trials Register (Registration number: DRKS00025291).

## Introduction

Similar to other European countries, more than 50% of the adult population in Germany is overweight (body mass index (BMI) between 25.0 and 29.9 kg/m^2^) and about a quarter suffers from obesity (BMI ≥ 30.0 kg/m^2^) [1]. Overweight and obesity are associated with multiple chronic diseases [2, 3] which can reduce life expectancy [4, 5]. Multimodal lifestyle interventions including an energy-reduced diet, an increase in physical activity, and behavioral modification are the first option for the treatment of overweight and obesity [6].

Besides traditional face-to-face settings, weight loss interventions delivered by smartphone applications (apps) are popular and increasingly used, but data on their effectiveness is still scarce and depends on the respective study design. A systematic review and meta-analysis by Villinger and colleagues [7] showed that nutrition apps (with functions like goal setting and self-monitoring) have a statistically significant effect on eating behavior and nutrition-related outcomes including BMI. However, it has to be mentioned that apps available within app stores have no access limitations and mainly include single functions and not a multimodal weight loss program as recommended by obesity guidelines [6].

On 19th December 2019, the concept of Digital Health Applications (DiHA) was introduced in the German healthcare system with the Digital Healthcare Act [8]. In contrast to lifestyle apps, DiHA are defined as medical devices (e.g. apps) supporting patient care regarding diseases (e.g. obesity), injuries, and disabilities. Health insurances cover all cost, that DiHA are free of charge for patients. For the access to a DiHA an activation key is needed which is provided if the patient proves a medical diagnosis (e.g. obesity) that matches the indication of the respective DiHA. The final certification of DiHA is conducted by the Federal Institute for Drugs and Medical Devices (BfArM, Bundesministerium für Arzneimittel und Medizinprodukte) and regulations require proof of a positive health care effect. This DiHA concept is unique and clearly distinguishes lifestyle apps from approved app-based programs.

The aim of this study was to evaluate the efficacy and to examine the positive health care effect of a digital multimodal lifestyle intervention in people with obesity provided by a DiHA. The primary objective was to evaluate the effect of the app on weight loss after 12 weeks of application. The secondary objective was to evaluate the effect of the app on body weight after 12 weeks of follow-up, to assess quality of life after 12 and 24 weeks, and to examine the usability and acceptance of the app.

## Methods

### Study design

This study was a single-centre randomized controlled trial that took place at the Institute for Nutritional Medicine at the School of Medicine & Health of the Technical University of Munich (TUM). The study protocol has been approved by the local ethics committee (vote number: 45/22 S-NP) and was registered at the German Clinical Trials Register (Registration number: DRKS00025291). All participants had given written informed consent before participation.

### Study population

Participants were recruited through social media (Facebook, Instagram), an advertising banner in the Munich subways, and flyers distributed to doctor’s offices in the Munich region between March 17th and August 9th 2022. Participants who met the following eligibility criteria were included into the study: adults (women, men), age between 18 and 70 years, BMI between 30.0 and 40.0 kg/m^2^, no severe diseases (e.g. diagnosed diabetes mellitus, cardiovascular disease, and cancer), ownership of a smartphone. Inclusion criteria were checked through a screening phone call and confirmed during the first study visit. Included participants were randomized to two study groups (ADHOC and EXPECT) with an allocation ratio of 1:1 using the Stat Trek Random Number Generator [9]. The trained study team which enrolled participants did not know the randomization list. Allocation was performed by the database after entering the participants’ data. Participants of both groups attended three study visits in total (baseline: V1, after 12 weeks: V2, after 24 weeks: V3) and received allowance of 25 € for V2 and V3 each.

### App-based intervention

The DiHA “Oviva Direkt für Adipositas” (Oviva AG, Potsdam, Germany) is available for iOS and Android and delivers a 12-week multimodal weight loss intervention program according to the German guidelines for the prevention and treatment of obesity [10]. In the beginning of the weight loss intervention (for ADHOC at V1, for EXPECT at V2 after 12 weeks of “waiting” period), participants were guided through the app installation by a member of the study team. In the first week of app use, participants received a phone call by a qualified coach employed by the app provider. This call aimed to ensure patients’ safety and the appropriate use of the medical device. Furthermore, the app included a private chatroom to ask questions if needed.

**Fig. 1:**
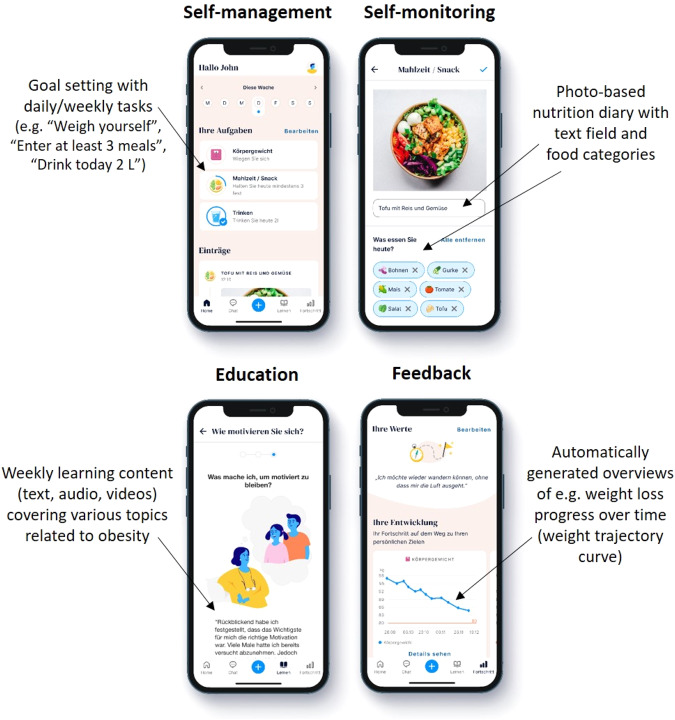
Screenshots of the study app.

The mode of action of the app-based program included three main elements: self-management, self-monitoring, and education (Fig. 1). As self-management approach, participants could set their daily/weekly goals by choosing ones suggested by the app or by choosing self-appointed ones. For self-monitoring purposes, participants could enter various data on e.g. nutrition, physical activity, and body weight. According to the data entered by the users, automated feedback was generated in form of weight trajectory curves, reminders (e.g. to enter the current weight at least once a week), motivating notifications, and interpretations (e.g. which food categories are under- or over-represented in the diet). For education, learning content (e.g. aetiology of obesity, lifestyle recommendations for weight loss, dealing with relapse) was provided on a weekly basis by text, audio, or video format. Regular learning success controls were offered. In summary, the weight loss program of the app uses different behavioural change techniques clustered by Michie et al. [11].

To mimic a real-world setting, participants in the ADHOC group did not get any further advices besides of using the app for 12 weeks. The ADHOC group received the app intervention in the first 12 weeks and was allowed to use the app for further 12 weeks of follow-up. The app was not uninstalled by the study team.

### Assessment of sociodemographic and anthropometric data and of quality of life

Sociodemographic data was collected at baseline through a standardized questionnaire. Anthropometrics (e.g. body weight, height, and composition) were objectively measured at all three study visits by the study team. Body height was measured to the nearest cm by using a stadiometer (SECA 214, Seca GmbH & Co., KG, Germany) and body weight and body composition was measured in light clothing, with an empty bladder, and without shoes by a bioimpedance analysis scale (BC-418MA, Tanita Europe B. V., Netherlands). The BMI has been calculated as body weight (in kg) divided by the square of body height (in m).

Health-related quality of life was assessed at all three study visits through the validated *EuroQol* (EQ-5D-5L) questionnaire [12] with five dimensions (mobility, self-care, usual activities, pain/discomfort, and anxiety/depression) and five response levels (no problems, slight problems, moderate problems, unable to/extreme problems) for each dimension. Responses were converted to an EQ-5D index value by using a standard EQ-5D-5L value set for Germany. The conversion was done in RStudio with the package “eq5d” by Morton Fraser [13]. In addition, the EQ-5D-5L includes a visual analogue scale from 0 (“The worst health you can imagine.”) to 100 (“The best health you can image.”) at which participants rated their current, subjective perception of health ( = EQ VAS score).

### Usability and acceptance

Data on app usability and user acceptance were collected after 12 weeks of app intervention. The two dimensions “perceived usefulness” and “perceived ease of use” (each represented by four items) of the validated questionnaire *Technology Acceptance Model 3* (TAM 3) [14] were used to calculate a total score. A higher total score means a more positive judgement of app usefulness and ease of use. By creating tertiles of the total score, completers were divided into three groups (“low” = assessment tends to be negative, “middle” = assessment is neither negative nor positive, “high” = assessment tends to be positive). To assess the app system usability, the validated questionnaire *System Usability Scale* (SUS) [15] was used. A higher SUS score stands for a more positive judgement of the app system usability. By creating tertiles of the SUS score, completers were divided into three groups (“low”, “middle”, and “high”).

Data on minutes spent on the app per week tracked by the app was used as a proxy for the intensity of app usage.

### Statistical analysis

The primary analysis population was all study participants providing weight data after 12 weeks of intervention ( = completers analysis). Integrity and plausibility checks were performed. Absolute and relative frequency, means, and standard deviations were calculated. For comparison of baseline characteristics between the groups, a Two sample *t*-test (for normally distributed outcomes) or a Mann-Whitney-*U* test (for non-normally distributed outcomes) was used. Normality was tested using the Shapiro-Wilk test and by graphical inspection of the distribution in each group. Variance homogeneity was checked by using Levene’s test. For categorical outcomes, Pearson’s chi-squared test or Fisher’s exact test was used. Multiple linear regression analysis adjusted for gender, age, and baseline body weight was conducted for comparison of changes after 12 weeks between the groups and for examining the association between time spent on the app and weight reduction. The 24 weeks data analysis was focussing on changes within the groups and not on group comparisons. For comparisons of the TAM and SUS scores, the Kruskal-Wallis rank sum test was conducted. *P*-values < 0.05 were considered as statistically significant. All statistical analyses were performed using RStudio (4.1.0). Assuming a 20% dropout, 156 participants are enough to show with a statistical power of 80%, a significance level of 0.05, and a standard deviation of 6%, a weight loss effect of 3% in the intervention group (ADHOC) which is statistically significant compared to the control group (EXPECT). The post-hoc power analysis with G*power [16] resulted in 99.9%.

For missing data on the primary outcome, last observation-carried forward (LOCF) imputation method was conducted. For the LOCF analysis after 12 weeks, the last weight tracked by the app was used as the last observation if measured weight was missing after 12 weeks. For the LOCF analysis after 24 weeks, the last measured body weight at the study centre and/or the last tracked weight by the app (self-reported) was used.

## Results

### Study profile

**Fig. 2:**
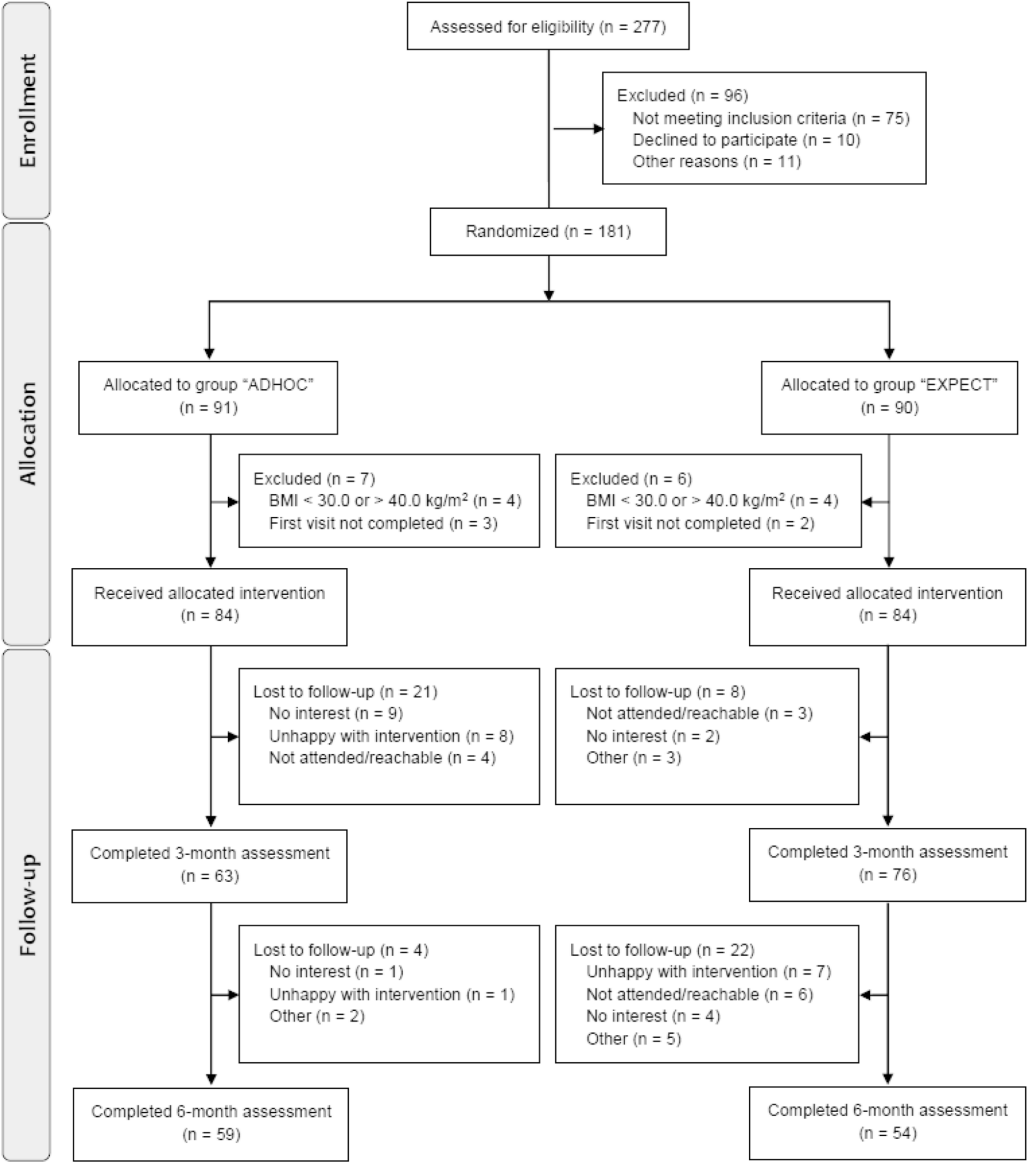
Study flow chart.

Figure 2 shows the study flow. From 277 interested participants assessed for eligibility, 181 were included into the study between March 17th (first patient in) and August 9th 2022 (last patient in). On February 14th 2023 was the last patient out (after 24 weeks of study duration).

### Baseline data

Population characteristics (*N* = 168) are presented in Table 1. Participants of the two groups did not differ significantly in any of the baseline parameters (all *p* > 0.05). For both groups together, the average age was 46.8 ± 11.0 years and mean BMI was 34.2 ± 2.8 kg/m^2^. The majority of participants were female (108/168, 64.3%), married (88/168, 52.4%), with university/college qualification (90/168, 53.6%), and working full-time (105/168, 62.5%).

**Table 1:** Baseline characteristics of total study participants (*N* = 168).

	ADHOC (*N* = 84)	EXPECT (*N* = 84)	*P*-value
	*n* (%) or mean ± SD	*n* (%) or mean ± SD	
Gender^a^			0.42
Female	57 (67.9)	51 (60.7)	
Male	27 (32.1)	33 (39.3)	
Age (years)^b^	47.4 ± 11.5	46.3 ± 10.6	0.50
<50	44 (52.4)	48 (57.1)	0.64
≥50	40 (47.6)	36 (42.9)	
Body weight (kg)^b^	100.8 ± 12.1	100.6 ± 14.8	0.93
Body height (cm)^c^	171.2 ± 8.9	171.5 ± 10.5	0.95
BMI (kg/m^2^)^b,d^	34.3 ± 2.5	34.1 ± 3.0	0.47
30.0–34.9	50 (59.5)	52 (61.9)	0.87
35.0–40.0	34 (40.5)	32 (38.1)	
Body composition^b^			
Fat mass (%)	39.3 ± 7.0	37.6 ± 8.1	0.18
Fat mass (kg)	39.4 ± 7.6	37.5 ± 8.6	0.12
Fat free mass (kg)	61.5 ± 11.8	63.1 ± 14.1	0.69
Marital status^c^			0.40
Single	28 (33.3)	33 (39.3)	
Married	47 (56.0)	41 (48.8)	
Divorced	7 (8.3)	10 (11.9)	
Widowed	2 (2.4)	0	
With partner in one household^a^			0.32
Yes	61 (72.6)	54 (64.3)	
No	23 (27.4)	30 (35.7)	
Education (years)^d^			0.32
8/9	5 (6.0)	8 (9.5)	
10	14 (16.7)	22 (26.2)	
12/13	14 (16.7)	14 (16.7)	
University/college	50 (59.5)	40 (47.6)	
Other	1 (1.2)	0	
Vocational education^d^			0.19
Yes	54 (64.3)	57 (67.9)	
No	28 (33.3)	27 (32.1)	
NA^e^	2 (2.4)	0	
Working^d^			0.99
Full-time	51 (60.7)	54 (64.3)	
Part-time	22 (26.2)	18 (21.4)	
Other	11 (13.1)	12 (14.3)	
Smoking status^d^			0.28
Yes (regularly/occasionally)	8 (9.5)	14 (16.7)	
Not anymore	34 (40.5)	25 (29.8)	
Never	42 (50.0)	45 (53.6)	
Comorbidities^d,f^			
Thyroid disease	13 (15.5)	16 (19.0)	0.68
Hypertension	14 (16.7)	11 (13.1)	0.67
Disease of the respiratory tract	11 (13.1)	8 (9.5)	0.63
Other^g^	52 (61.9)	35 (41.7)	all > 0.05
Quality of life^h^			
EQ-5D-5L index (≤1)	0.94 ± 0.1	0.96 ± 0.1	0.33
EQ VAS score (0–100)	72.6 ± 14.3	76.6 ± 12.4	0.06

^a^Categorical data, Pearson’s Chi-squared test used for comparison.

^b^Normally distributed, Two sample *t*-test used for comparison.

^c^Not normally distributed, Mann-Whitney-*U* test used for comparison.

^d^Categorical data, Fisher’s exact test used for comparison.

^e^Not available.

^f^Multiple answers possible.

^g^Other comorbidities summarized: hypercholesterolemia, sleep apnea, disc prolapse, etc.

^h^Not normally distributed, Mann-Whitney-*U* test used for comparison.

### Weight change and quality of life data after 12 and 24 weeks

In total, 82.7% (139/168) of the participants completed the visit after 12 weeks (V2), 75.0% (63/84) in the ADHOC group and 90.5% (76/84) in the EXPECT group (Fig. 2). In the completers analysis, the participants in the ADHOC group showed a mean weight loss of 3.2 ± 3.2 kg (3.2 ± 3.0% of their baseline body weight), whereas weight loss in the EXPECT group was 0.4 ± 2.6 kg (0.3 ± 2.6%) with a significant difference of 2.9 kg (95% CI: [−3.8; −1.9], *p* < 0.001, standardized regression coefficient β = 0.45) or 2.9% (95% CI: [−3.8; −1.9], *p* < 0.001, β = 0.46) (Table 2). Likewise, the mean changes of body composition were significantly different between the two groups (all *p* < 0.01) (Table 2). The ADHOC group showed a higher mean fat mass loss with 2.0 ± 2.7 kg compared to the EXPECT group with 0.1 ± 2.2 kg (*p* < 0.001).

**Table 2:** Completers and LOCF imputation analysis after 12 weeks and 24 weeks.

Completers	Change after 12 weeks (*N* = 139)					Change after 24 weeks (*N* = 113)	
	ADHOC (*N* = 63)	EXPECT (*N* = 76)	Mean difference [95% CI]^b^	*P*-value	Effect size^c^	ADHOC (*N* = 59)	EXPECT (*N* = 54)
	mean ± SD or *n* (%)	mean ± SD or *n* (%)				mean ± SD or *n* (%)	mean ± SD or *n* (%)
Weight change (kg)	−3.2 ± 3.2	−0.4 ± 2.6	−2.9 [−3.8; −1.9]	< 0.001	0.45	−3.3 ± 4.8	−2.1 ± 3.3
Weight change (%)	−3.2 ± 3.0	−0.3 ± 2.6	−2.9 [−3.8; −1.9]	< 0.001	0.46	−3.1 ± 4.5	−2.0 ± 3.3
< −3.0	20 (31.7)	35 (46.1)				19 (32.2)	14 (25.9)
≥ −3.0	32 (50.8)	9 (11.8)				25 (42.4)	22 (40.7)
from that ≥ −5.0	18 (28.6)	4 (5.3)				15 (25.4)	6 (11.1)
No weight loss	11 (17.5)	32 (42.1)				15 (25.4)	18 (33.3)
Change in body composition^a^							
Fat mass (%)	−0.8 ± 1.9	+ 0.04 ± 1.7	−0.8 [−1.4; −0.2]	0.008	0.23	−0.05 ± 2.5	+ 0.6 ± 1.9
Fat mass (kg)	−2.0 ± 2.7	−0.1 ± 2.2	−1.8 [−2.6; −1.0]	< 0.001	0.34	−1.3 ± 4.0	−0.03 ± 2.5
Fat free mass (kg)	−1.3 ± 1.7	−0.3 ± 2.0	−1.0 [−1.7; −0.4]	0.002	0.27	−2.2 ± 1.9	−1.9 ± 1.8
Change in quality of life							
EQ-5D-5L index	−0.02 ± 0.1	−0.001 ± 0.1	−0.02 [−0.05; 0.01]	0.32	-	+ 0.02 ± 0.1	+ 0.002 ± 0.1
EQ VAS score	+ 4.7 ± 14.8	+ 0.6 ± 11.0	+ 4.3 [−0.1; 8.6]	0.06	-	+ 7.7 ± 13.3	+ 4.4 ± 15.1

LOCF imputation	Change after 12 weeks (*N* = 168)					Change after 24 weeks (*N* = 168)	
	ADHOC (*N* = 84)	EXPECT (*N* = 84)	Mean difference [95% CI]^b^	*P*-value	Effect size^c^	ADHOC (*N* = 84)	EXPECT (*N* = 84)
	mean ± SD or *n* (%)	mean ± SD or *n* (%)				mean ± SD or *n* (%)	mean ± SD or *n* (%)
Weight change (kg)	−2.6 ± 3.1	−0.3 ± 2.5	−2.3 [−3.1; - 1.4]	< 0.001	0.38	−2.4 ± 4.2	−1.4 ± 3.1
Weight change (%)	−2.6 ± 3.0	−0.3 ± 2.5	−2.3 [−3.2; - 1.5]	< 0.001	0.39	−2.4 ± 4.0	−1.3 ± 3.1
< −3.0	27 (32.1)	35 (41.7)				21 (25.0)	22 (26.2)
≥ −3.0	25 (29.8)	9 (10.7)				27 (32.1)	26 (31.0)
from that ≥ −5.0	20 (23.8)	4 (4.8)				16 (19.0)	7 (8.3)
No weight loss	22 (26.2)	40 (47.6)				36 (42.9)	36 (42.9)

^a^After 12 weeks: *N* (EXPECT) = 75; after 24 weeks: *N* (ADHOC) = 53, *N* (EXPECT) = 51.

^b^Results are presented as unstandardized regression coefficients adjusted for gender, age, and baseline body weight.

^c^Results are presented as standardized regression coefficients adjusted for gender, age, and baseline body weight.

Regarding quality of life, the mean change of the EQ-5D-5L index (−0.02 ± 0.1 vs. −0.001 ± 0.1, *p* = 0.32) and the EQ VAS score (+4.7 ± 14.8 vs. + 0.6 ± 11.0, *p* = 0.06) was not significantly different between the two groups (Table 2). The 12 weeks follow-up visit (V3) was completed by 67.3% (113/168) of participants resulting in a study adherence rate of 70.2% (59/84) in the ADHOC group and 64.3% (54/84) in the EXPECT group (Fig. 2).

After 24 weeks, weight maintenance was observed with a mean weight loss from baseline of 3.3 ± 4.8 kg (3.1 ± 4.5%) in the ADHOC group (Table 2).

After LOCF imputation, mean weight loss after 12 weeks was 2.6 ± 3.1 kg (2.6 ± 3.0%) in the ADHOC group and 0.3 ± 2.5 kg (0.3 ± 2.5%) in the EXPECT group with a significant difference of −2.3 kg (95% CI: [−3.1; −1.4], *p* < 0.001, β = 0.38, Table 2) or −2.3% (95% CI: [−3.2; −1.5], *p* < 0.001, β = 0.39, Table 2) between the groups.

Sensitivity analyses by gender, age groups (<50 and ≥50 years), and BMI groups (30.0–34.9 and 35.0–40.0 kg/m^2^) confirmed a statistically significant higher weight loss after 12 weeks of intervention in the ADHOC group compared to the EXPECT group (all *p* < 0.001, *data not shown*). Men lost more weight than women (ADHOC: −4.1 ± 4.0 kg vs. −2.8 ± 2.6 kg), participants older than 50 years lost more weight than participants younger than 50 years (ADHOC: −3.7 ± 3.5 kg vs. −2.7 ± 2.8 kg), and participants with a BMI of 35.0 to 40.0 kg/m^2^ lost more weight than participants with a BMI of 30.0 to 34.9 kg/m^2^ (ADHOC: −3.8 ± 3.9 kg vs. −2.9 ± 2.6 kg).

### App use and association with weight loss

In the ADHOC group, a mean TAM score of 5.4 ± 1.1 and a mean SUS score of 76.2 ± 18.5 after 12 weeks of app intervention was found (*data not shown*). It could be shown that among completers of the ADHOC group weight loss success after 12 weeks of app intervention significantly differed between the three TAM groups (*p* = 0.003) (Table 3). Pairwise comparison resulted in a significantly greater weight loss in ADHOC participants scoring middle or high in TAM compared to those with low scoring (middle vs. low: −4.6 ± 3.4 kg vs. −1.5 ± 2.8 kg, *p* = 0.004; high vs. low: −3.8 ± 2.6 kg vs. −1.5 ± 2.8 kg, *p* = 0.025, *data not shown*). No significant difference in weight loss was found between the three SUS groups (*p* = 0.16) (Table 3).

**Table 3:** Descriptive analysis of completers in both groups on basis of the TAM and SUS score.

ADHOC								
	TAM^a^ (*N* = 63)				SUS^b^ (*N* = 63)			
	Low (*N* = 21)	Middle (*N* = 21)	High (*N* = 21)	*P*-value	Low (*N* = 21)	Middle (*N* = 21)	High (*N* = 21)	*P*-value
**Weight loss (kg)**^**c**^	−1.5 ± 2.8	−4.6 ± 3.4	−3.6 ± 2.4	0.003	−2.2 ± 2.8	−3.9 ± 3.9	−3.6 ± 2.4	0.16
**Weight loss (%)**^**c**^	−1.4 ± 2.7	−4.4 ± 2.9	−3.8 ± 2.6	0.002	−2.2 ± 2.8	−3.7 ± 3.5	−3.7 ± 2.5	0.17
< −3.0	8 (38.1)	6 (28.6)	6 (28.6)		9 (42.9)	5 (23.8)	6 (28.6)	
≥ −3.0	5 (23.8)	14 (66.7)	13 (61.9)		7 (33.3)	12 (57.1)	13 (61.9)	
from that ≥ −5.0	3 (14.3)	7 (33.3)	8 (38.1)		4 (19.0)	6 (28.6)	8 (38.1)	
No weight loss	8 (38.1)	1 (4.8)	2 (9.5)		5 (23.8)	4 (19.0)	2 (9.5)	

EXPECT								
	TAM^d^ (*N* = 54)				SUS^e^ (*N* = 54)			
	Low (*N* = 18)	Middle (*N* = 18)	High (*N* = 18)	*P*-value	Low (*N* = 18)	Middle (*N* = 18)	High (*N* = 18)	*P*-value
**Weight loss (kg)**^**c**^	−1.1 ± 2.6	−0.9 ± 2.1	−2.8 ± 2.3	0.03	−1.4 ± 2.5	−1.1 ± 2.3	−2.3 ± 2.5	0.29
**Weight loss (%)**^**c**^	−1.0 ± 2.6	−0.9 ± 2.2	−2.8 ± 2.2	0.04	−1.2 ± 2.5	−1.2 ± 2.5	−2.3 ± 2.4	0.37
< −3.0	6 (33.3)	8 (44.4)	6 (33.3)		5 (27.8)	6 (33.3)	9 (50.0)	
≥ −3.0	4 (22.2)	3 (16.7)	10 (55.6)		5 (27.8)	5 (27.8)	7 (38.9)	
from that ≥ −5.0	2 (11.1)	1 (5.6)	3 (16.7)		2 (11.1)	1 (5.6)	3 (16.7)	
No weight loss	8 (44.4)	7 (38.9)	2 (11.1)		8 (44.4)	7 (38.9)	2 (11.1)	

^a^Mean ± SD: Low = 4.0 ± 0.9; Middle = 5.7 ± 0.3; High = 6.4 ± 0.2.

^b^Mean ± SD: Low = 55.2 ± 16.4; Middle = 81.1 ± 4.4; High = 92.4 ± 3.4.

^c^Not normally distributed, Kruskal-Wallis-test used for the comparison.

^d^Mean ± SD: Low = 3.8 ± 0.7; Middle = 5.2 ± 0.2; High = 6.2 ± 0.3.

^e^Mean ± SD: Low = 56.5 ± 9.8; Middle = 76.4 ± 4.6; High = 89.7 ± 4.8.

After 12 weeks of app intervention, the EXPECT group had a mean TAM score of 5.1 ± 1.1 and a mean SUS score of 74.2 ± 15.3 (*data not shown*). A significant difference in weight loss was found between the three TAM groups (*p* = 0.03), but not after pairwise comparison (all *p* > 0.05). No statistically significant difference in weight loss was found between the three SUS groups (*p* = 0.29) (Table 3).

Multiple linear regression analysis adjusted for gender, age, and baseline body weight resulted in an association between TAM score and weight reduction (β [95% CI] = −1.7 [−2.7; −0.7], *p* = 0.002, *data not shown*). No association between SUS score and weight reduction could be shown (*p* = 0.11). In the EXPECT group no association at all could be found (all *p* > 0.05, *data not shown*).

**Fig. 3:**
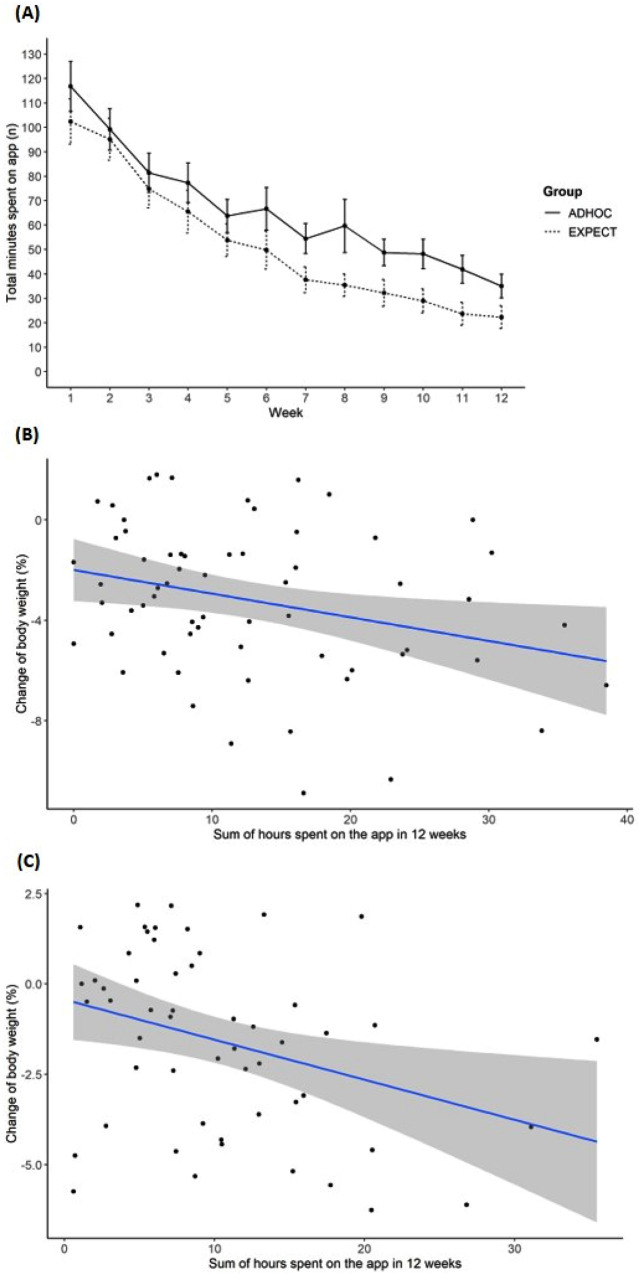
Time spent on the app by completers. Mean number of minutes spent on the app of completers per week and group (+SEM) (**A**) and relationship between time of app use over 12 weeks and weight change in the ADHOC (**B**) and EXPECT group (**C**).

In both groups the mean number of total minutes spent on the app per week decreased from week one to week 12 during the app intervention phase (Fig. 3A).

Multiple linear regression analysis was conducted to examine the association of time of app use (in hours) over 12 weeks and weight reduction per group. For completers of the ADHOC group, the model showed that an increasing sum of hours spent on the app within 12 weeks of intervention was associated with an increased weight reduction (β [95% CI] = −0.10 [−0.18; −0.01], *p* = 0.03) (Fig. 3B). For completers of the EXPECT group, a similar result was observed (β [95% CI] = −0.12 [−0.22; −0.02], *p* = 0.02) (Fig. 3C).

## Discussion

The results of this app-based multimodal weight loss program showed a statistically significant weight and fat mass loss after 12 weeks and consecutive weight maintenance after 12 weeks of follow-up. Furthermore, it became evident that the extent of weight loss was dependent on the perceived app usability and user acceptance as well as on the duration of app use.

The observed weight loss of 2.6% (LOCF) and 3.2% (completers analysis), respectively, within 12 weeks was moderate and comparable to another study in which an app intervention in combination with a smart band to track physical activity resulted in a loss of body weight of 1.97 kg after 3 months [17]. Several studies have confirmed that moderate weight loss has beneficial effects on various outcomes. It was reported that a weight loss of 1 kg improves systolic blood pressure by 1.1 mmHg and diastolic blood pressure by 0.9 mmHG [18], or decreases fasting blood glucose by 4 mg/dl (0.2 mmol/l), respectively [19]. A pooled analysis of more than 2,000 participants including 25% men, at a mean age of 50 years and a mean BMI of 32.7 kg/m^2^ revealed that each kilogram weight loss was associated with a reduction of systolic/diastolic blood pressure by 0.4 mmHg/0.3 mmHg and a reduction of HbA1c by 0.2 mmol/mol [20].

In this study, the app was used for 12 weeks due to a specific regulation in Germany where DiHA are prescribed for a quarter, with the option for an extension. Therefore, the 12-week app intervention period was followed by a voluntary 12-week extension of app use, which did not result in further weight loss, but the reduced body weight was maintained. A period of 12 weeks of app use may be too short to fully exploit the potential effect on body weight, since maximum weight reduction is usually observed after four to six months or even later, depending on the type and modalities of a lifestyle program [21, 22]. This may indicate that a longer app usage may be recommendable to achieve a greater weight loss. For instance, in a study with three intervention arms, the group receiving a smartphone-based treatment in combination with online lessons, feedback, and monthly weigh-ins showed a mean weight loss of 5.5 kg (95% CI: 3.9; 7.0) after 18 months [23]. In a recent German RCT evaluating an app-based multimodal weight loss program in adults with obesity, a mean self-reported weight loss of 8.0% (95% CI: −9.89; −6.01) based on completers and 7.8% (95% CI: −9.66; −5.84) based on the intention-to-treat analysis was observed after 12 months of intervention [24].

To date, the literature on mHealth approaches for the treatment of obesity is heterogeneous, as multiple combinations of app-based interventions with other tools are frequently applied. Usually, the application of a solely app-based lifestyle intervention results in moderate weight loss over 24 months [25]. In the “EVIDENT 3” trial with a total of 440 participants that evaluated the effectiveness of a multicomponent mobile health intervention including an app, an activity tracker wristband, and brief counselling, the intervention group showed a greater weight loss after three months (−1.97 kg, 95% CI −2.39 to −1.54) relative to the control group (−1.13 kg, 95% CI −1.56 to −0.69 kg) according to the completers analysis [17].

Despite the moderate weight loss, solely app-based interventions have the advantage of a high scalability because they can be easily provided to the large number of people with obesity. In the German healthcare system, DiHA can be prescribed for people with obesity as the only basic intervention program which is free of charge for patients. Dependent on obesity grade and on obesity-related comorbidities a subgroup of patient needs a more individual and intense clinical care.

In terms of app usage and acceptance both groups had similar TAM scores after 12 weeks of intervention. Interestingly, participants of the ADHOC group with a middle or high TAM score showed a significantly greater weight loss compared to participants with a low TAM score indicating that a more positive attitude toward app usability and user acceptance was associated with a greater weight reduction. Therefore, the use of such questionnaires might be helpful for early identification of individuals who would particularly benefit from app use or probably not because of e.g. limited technology acceptance. A systematic review summarized the factors influencing users´ acceptance and use of apps. It shows, that a lack of fundamental motivation to alter the current situation can limit the efficacy and acceptance of even the most-well-designed app [26].

App user adherence, measured here as minutes spent on the app per week decreased over time, which is in line with other studies [27–29]. This continuous decrease can be attributed to a saturation effect, meaning that individuals tend not to maintain a behaviour or activity because the direct benefit decreases over time [30]. Another possibility could be a difference in engagement and effectiveness because of different sociodemographic factors like gender, age, and education as shown in a systematic review including 13 mobile health intervention studies [31]. Nevertheless, it could be shown for both groups that participants spending more time on the app, and probably having a greater benefit from the used behavioural change techniques, had a greater weight reduction compared to those spending less time on the app. A systematic review showed that self-monitoring is associated with weight loss [32].

Real-world-data provided by a commercial app provider and containing data from 25,706 persons (United Kingdom, Germany, Switzerland) who used an app for prevention and therapy of nutrition-related conditions could identify a relationship between the app components and weight change [33]. Self-management, self-monitoring, learning time, and coaching were positively associated with weight loss [33]. These effective app features were also mainly included in the DiHA investigated in this study. It is assumed, that the unique combination of an evidence-based multimodal weight loss program with various behavioural change techniques fully remotely delivered by the here investigated app is key element for the weight loss effect in this study. Future weight loss apps should consider this approach and should not focus on single components. This is in line with the complex nature of the aetiology of obesity.

A limitation of the study is that it isn’t representative for the population in Germany. In addition, it was conducted as a single-centre study in the Munich region. Compared to other weight loss studies, the gender distribution showed a rather high proportion of men compared to women (35.7% vs. 64.3%). Furthermore, all age groups and the pre-defined BMI range (30.0 to 40.0 kg/m^2^) are represented by this study sample. A strength of this study in comparison to other studies was that body weight was objectively assessed during three visits by a trained study team. Furthermore, it should be emphasized that in this study support was provided fully remote without any kind of therapeutic human contact. The app was used in a self-guided way and therefore, this is one of the first studies including a fully digital weight loss intervention.

## Conclusion

The findings of this intervention study demonstrate a clinically meaningful weight loss in people with obesity by a fully digital lifestyle intervention program. The moderate weight loss within the first 12 weeks was maintained in the 12-week follow-up period. Overall, the results suggest that this multimodal app-based weight loss program may be an effective tool prescribed by physicians and psychotherapists for initial weight loss in the general population with obesity.

## Data Availability

The datasets used and/or analysed and the computer codes are available from the corresponding author upon reasonable request.
